# Treatment of Diabetic Foot Ulcers in the Home: Video Consultations as an Alternative to Outpatient Hospital Care

**DOI:** 10.1155/2008/132890

**Published:** 2008-02-13

**Authors:** Jane Clemensen, Simon B. Larsen, Marit Kirkevold, Niels Ejskjaer

**Affiliations:** University of Aarhus, Nordre Ringgade 1, C 8000 Aarhus, Denmark

## Abstract

The aim of this study was to investigate whether video consultations in the home can support a viable alternative to visits to the hospital outpatient clinic for patients with diabetic foot ulcers. And furthermore whether patients, relatives, visiting nurses, and experts at the hospital will experience satisfaction and increased confidence with this new course of treatment. Participatory design methods were applied as well as field observations, semistructured interviews, focus groups, and qualitative analysis of transcriptions of telemedical consultations conducted during a pilot test. This study shows that it is possible for experts at the hospital to conduct clinical examinations and decision making at a distance, in close cooperation with the visiting nurse and the patient. The visiting nurse experienced increased confidence with the treatment of the foot ulcer and characterized the consultations as a learning situation. All patients expressed satisfaction and felt confidence with this new way of working.

## 1. INTRODUCTION

Diabetes, late complications hereof, and diabetic foot ulcers in particular present increasing challenges in terms of amputations, sick leaves, reduced functioning, and escalating financial costs [1]. The patients' quality of life decreases dramatically with the occurrence of diabetic foot ulcers. Currently, most treatment is carried out by nonexperts such as visiting nurses and general practitioners [2, 3] despite the fact that studies demonstrate the importance of specialized knowledge in the treatment of chronic wounds [3, 4]. To enable the experts to monitor an increasing number of patients more regularly, telemedicine (telemedicine, in its broadest sense, means “delivery of healthcare and the exchange of healthcare information across distances” [5]) has been suggested as a way of creating alternative treatment methods [6].

Previous studies have documented coherence between expert assessments of ulcers based on digital images versus live assessment [7, 8]. Even low-resolution images may sometimes be sufficient [9, 10]. Digital photographs have also proven valuable in learning situations, for example, when giving directions for wound care [11]. Only a preliminary set of experiences exists in using the emerging UMTS (universal mobile telephony system) technology for telemedicine, for example, for teleconsultations from a moving ambulance [12], but so far the results are promising.

Conclusions from other studies demonstrate that expert-guided treatment makes nurses use more advanced wound care remedies with better healing outcomes as a result [2, 3] and it leads to increased patient satisfaction through less travel and reduced waiting time [13–15]. Dansky and Bowles have even suggested that communicating with the expert over a distance facilitates a more optimal dialog, which empowers the patients who participate more actively in their care planning. This new communication form creates a new bond—a relationship based on partnership.

Furthermore, Dansky and Bowles found a significant learning curve for nurses and expect that the nurses will become increasingly comfortable and efficient as the technology becomes pervasive [16–18]. Another outcome of the Kaiser Permanente Tele-Home Health Research Project was that the patients became more actively involved in attending their own health care needs and the remote visits were characterized as convenient because the patients felt comfortable with discussing personal problems and in receiving care at home [14]. Treatment of diabetic ulcers has turned out to be applicable to both synchronous [19] and asynchronous telemedicines [20]. Willbright found that telemedicine made the specialist nurse more empowered and enabled to independently develop treatment plans [19].

Most of these studies have utilized a static installation in the patient's home or a store-and-forward solution for enabling the visiting nurse to receive advice from experts. Thus, the telemedical solutions have either connected the patient with the visiting nurse, or the visiting nurse with the expert. To a large extent, the treatment can be perceived as collaboration between these three stakeholders (the patient, the visiting nurse, and the expert). For this reason, enabling the three partners to be simultaneously engaged in the communication taking place may increase the extent to which the expert knowledge can be utilized directly in the treatment. Furthermore, a direct dialog between people with different perspectives may enable a more holistic view and thereby result in a more tailored treatment.

To pursue this goal, we investigated the technological possibilities for supporting a new organization of the treatment where the expert, the visiting nurse, and the patient in collaboration could discuss the situation and plan the treatment.

In this article, we present the results of a pilot test, the aim of which was to investigate the possibility of having the expert participating in the home consultations by the use of technology and in cooperation with the visiting nurse and the patient. In this article, we define “experts” as either a nurse or a doctor specialized in treatment of diabetic foot ulcers in our study located at Aarhus Hospital, Denmark. The visiting nurses referred to in this article are associated with local community centers in the residential area of the respective patients and they were nonexperts in diabetic foot ulcers.

We explore the subjective satisfaction of patients and healthcare professionals to test the hypothesis that a telemedical consultation can be a viable alternative to a visit to the outpatient clinic. In order for a telemedical consultation to be characterized as a viable alternative to a visit to the outpatient clinic, all participants must experience the telemedical treatment as satisfactory in terms of (1) the clinicians being provided with sufficient clinical information for clinical assessments and decisions to be made accurately, (2) the visiting nurses feeling supported and secured during the sessions, and (3) the patient being satisfied with the consultations. In the following, the findings related to these three issues are presented.

## 2. METHODS

During a 2-year process of participatory design, initial problems and potential solutions in the field of treatment of diabetic foot ulcers were investigated. Participatory design (PD) is a research approach, derived from action-based research that is applied to the development of technological and organizational solutions to real-world problems [21, 22]. PD is a qualitative study, in which the aim is to have close cooperation with the users during the process in order to ensure that the (technological) result can be understood and handled in practice by the users [23]. In our study, the group of participants consisted of hospital doctors and nurses specialized in diabetic foot ulcers, visiting nurses, patients, and relatives. The PD process contained field studies, workshops with the users, experiments in both laboratories and hospitals, and specification of an electronic ulcer record. The details of the methodology are described in another article [24]. The PD process resulted in the creation of a telemedical system for supporting the pilot test carried out during summer 2005. This article reports the results of the test.

### 2.1. Setup for the pilot test intervention

Five patients who were all mentally well functioning and following a course of treatment in the outpatient clinic were selected for inclusion by the experts—the same hospital clinicians who had participated in the PD process (1 doctor and 3 nurses). After the patients gave informed consent (including permission to film the video consultations), we contacted the management of the patient's local center to inform about the study and to obtain permission to involve the patient's visiting nurse. After that, we informed the visiting nurse in more detail and a meeting was set up for demonstration of the technology. The project was approved by the Regional Ethics Committee.

The patients were offered 3 consecutive video consultations to substitute 3 visits to the outpatient clinic all conducted by the same team of clinicians. A telemedical course of treatment was initiated by an intensive consultation in the outpatient clinic, where the experts created the patient file in the online ulcer record and uploaded high-resolution images of the ulcer as well as obtaining measurements such as peripheral blood pressure, blood sugar. Furthermore, the first of the three telemedical consultations was prescheduled by the researchers with both the visiting nurse, the patient and the hospital experts.

**Figure 1:**
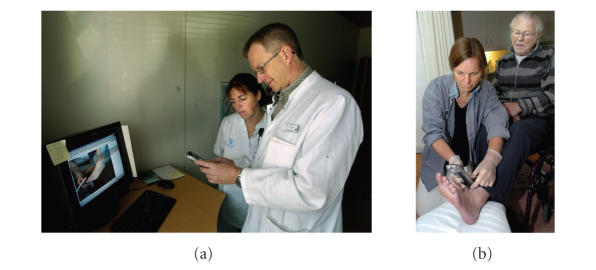
A video consultation from the pilot test. (a) The doctor and expert nurse at the hospital. (b) The visiting nurse and the patient at the home. The pictures originate from an article in a Danish newspaper (Morgenavisen JyllandsPosten, 10th May 2005) describing the project and the patient in picture on the right has given permission to its publication.

The telemedical setup consisted of UMTS video telephones (Motorola A920) and a custom designed online ulcer record. Prior to the consultations, the visiting nurse used the video phone to take pictures of the ulcer and transmit them to the ulcer record. As the video phones currently available on the market may not yet provide a sufficient image quality, the images from the phone were supplemented with images from a standard digital camera (Nikon CoolPix5400). These high-resolution images were uploaded to the ulcer record upon return to the office and therefore not available during the online consultations, but they were used as a safety precaution. (The experts were able to view those pictures later in cases of uncertainty. No changes were made to the assessment or the treatment on the background of these images, however.) At the agreed time of consultation, the visiting nurse made a video call from the home to the doctor and to an expert nurse at the hospital. The experts used (1) the still images and the video stream from the video phones, (2) images from the previous consultation, and (3) the cooperation of the visiting nurse in order to evaluate the ulcer and to prescribe further treatment. After the consultation, the experts wrote their summary of the consultation in the online web-based record. Figure 1 shows two photographs from one of the consultations.

### 2.2. Data collection

Each consultation was video filmed by the researchers both in the patient's home and at the hospital and transcribed afterwards. After completion of the telemedical experiments, the researchers conducted semistructured interviews of one-hour duration with each of the 5 visiting nurses at their local center and with the patients in their homes (which also involved a relative in 3 out of the 5 cases). Furthermore, we conducted a focus group interview with the 3 expert nurses to gain a guided discussion (all expert nurses had been informally interviewed individually after each consultation) and one individual semistructured interview with the doctor. As a final measure to circumvent amicable bias due to the personal presence of the researchers in the interviews, all participants were asked to indicate their satisfaction with every consultation in a questionnaire.

The total data material consisted of transcripts of field studies, workshops, experiments, interviews, and video consultations as well as the questionnaires and a specification of the electronic ulcer record. The material was analyzed systematically where data were categorized in themes arising during the analysis, both concerning the participants’ satisfaction, as described in this article as well as themes described in other articles elsewhere.

## 3. RESULTS

The 15 consultations were performed as planned. Using UMTS for telemedicine was a challenge. Various technical difficulties arose as described in another article [25]. The technical difficulties did not, however, prevent the pilot test to be carried out as planned, and the clinical results of the test are presented in this article. A wide range of clinical situations were encountered ranging from a noncritical healing ulcer to more complicated ulcers, and at one consultation the decision was a hospital admission. In another case, a patient was referred to the outpatient clinic for face-to-face specialist evaluation; however, the clinical conclusion based on the telemedical consultation remained unchanged. The consultations lasted from 5 to 18 minutes, including the dialogs between nurses and patients and also including scheduling a time for the ensuing video consultation. The course of 3 following consultations for each patient lasted from 2 weeks to 6 weeks.

The overall results from the study demonstrate that the alternate course of treatment proved satisfactory from the viewpoint of all 3 stakeholders. The experts were both satisfied with the new course of treatment and they felt secure in being responsible for the treatment performed on a distance. The visiting nurses were satisfied and felt supported in carrying out the treatment. The patients (and relatives) felt secure and characterized the treatment as an improvement because they were able to remain at home.

### 3.1. Expert’s basis for making decisions at a distance

After the consultations, the experts expressed satisfaction with the clinical basis for their decision making. Both the information provided in the form of pictures and communication with the patient and the cooperation with the visiting nurse constituted important basis for the experts' decision making. “The presence of the visiting nurse in the patient’s home compensates in full for not having the patient in front of you,” the doctor said in the interview.

By cooperating with the visiting nurse and being precise when asking questions, the experts were able to gather the information they would normally observe themselves, including the color, temperature, and smell of the foot ulcer. The high-resolution images from the digital camera were “even better than the human eye as they compared in quality to using a magnifying glass,” explained the doctor in the interview. However, as these pictures were uploaded following each consultation, the experts had only access to high-resolution images from the previous consultations at the time of the consultation. For this reason, the less optimal images from the video phones provided the main basis for the decisions, and if these images were not optimal in terms of, for example, lightning or sharpness, the visiting nurse observations were particularly useful. The possibility of comparing pictures of an ulcer from one visit to the next also supported the assessment of healing progress.

An important decision to make for the experts was how much responsibility could be delegated to the visiting nurse. The expert nurses had the possibility to get a feeling of the visiting nurses skills and if extra help was needed in carrying out the prescribed treatment. The expert nurse noted that she could value the visiting nurses level of competences just by looking at the way she handled the instruments. These observations lead the expert nurse towards what kind of instruction the visiting nurses needed or what she was able to ask from her. The doctor also assessed the competence of the visiting nurse, and described it in this way: “I’m used to asking 3 questions and then I can almost grade them (the visiting nurses).”

In the beginning of the pilot test, the roles of the participants in the telemedical consultations were not defined prior to each consultation. During the pilot tests, a firmer structure for the consultation gradually evolved. For instance, the experts became more aware of the fact that they had to combine their own views with that of the visiting nurse, and that the consultations were most efficient if one person took the lead. For these reasons, the experts made it a practice to hold a preconsultation to coordinate their impression before hearing the visiting nurse. They were conscious, however, not to put forward their impression at the actual consultation until they had heard what the visiting nurse had to say in order to be able to combine their own impression from the images with the subjective opinion of the visiting nurse. Furthermore, the ongoing dialog—once connected—brings forth issues that have an impact on the decisions, for instance, aspects of the patient’s daily living. The direct dialog with the visiting nurse provides the expert with a more detailed knowledge about the patient and the capabilities of the patient’s surroundings (e.g., the service levels at the local center). These issues would have been more difficult to pursue in a hospital setting, where the knowledge of the visiting nurse is absent. Consequently, in terms of the expert’s basis for decisions, the video consultation provided a feasible alternative to a visit in the outpatient clinic; and in some cases, the decision basis may even be more holistic.

### 3.2. Support and satisfaction for the visiting nurses

The visiting nurses were all positive towards the video consultations, which is in line with previous studies (see, e.g., [26]). They felt professionally supported in carrying out the treatment. Four out of five visiting nurses emphasized that participating in the project had increased their professional skills, by receiving information and instructions from the hospital. In contrast to the situation today, the direct contact with the experts means that a treatment plan may be decided and any discrepancies sorted out immediately. The visiting nurse is also instructed about new treatments which he/she understands more readily on the basis of expert reflections and explanations.

In this way, the dialog facilitates a decision process based on mutual understanding rather than the expert giving direct instructions. This ensures that the visiting nurse can relate to the treatment plan, which promotes satisfaction with the cooperation. In the interview, this particular visiting nurse was asked, if she at times took offence by receiving instructions on her professional field. She answered: “No, I think there has been room for me to express my opinions *…* If I had had a different opinion I would have had the possibility for discussing it. I feel there was two-way communication.”

The visiting nurses also agreed that the treatment procedures would improve when working in a team with the experts. As one nurse explained: “When I work on my own, I sometimes doubt if the treatment is sufficient, or if a doctor should see the ulcer. To be on the safe side I sometimes send the patient to the hospital, but when the patient returns, it is still the same treatment that was prescribed in the first place. This means that I misjudged the situation—that would not happen with this set-up.” The visiting nurse became the central coordinating actor combining the two roles as the “patient’s advocate” and the expert’s “prolonged arm.” One nurse quoted: “With the doctor I find out which treatment is necessary, and with the patient I find out if that is what we need. *…* It (her role) is more “here and now,” more present. It is a good role to have.” The collaboration facilitated a synergetic combination of the actors’ individual competences. “What they need on the hospital is what the visiting nurse can see in the home” (quote VN). Furthermore, the nurses expressed that the knowledge attained through the cooperation with the experts would also benefit their other patients. In this sense, the telemedical organization could enable more tailored treatment for the individual patient, which ultimately may increase the quality of the treatment.

### 3.3. Patient satisfaction

The patients expressed that being able to stay at home during treatment was a great advantage. In consistency with other studies [13, 27], patients complain about spending many hours waiting for transportation to the hospital, waiting in the outpatient clinic, and again waiting for transportation home.

The video consultation focused not just on the ulcer, but included the patient as well. When the consultation is performed in the patient’s home, patients are more self-confident and more apt to ask about personal issues, such as sleeping problems, pain, medication [17]. One patient quoted: “I find it reassuring—it is not always that you get to ask the questions you have when you go up there (to the hospital)—it is easier when you are in your home.” Another patient described the consultation in the following way: “The contact between the visiting nurse and the experts is good. They work as a team.” By having access to the expert and the visiting nurse at the same time, the patients are able to receive immediate answers to their questions and at the same time follow the visiting nurse getting instructions from the expert.

The online dialog was accepted by the patients: “It is easier to make contact, when there is a picture, in which you can see the person instead of only a phone. I felt safe. I think the doctor cared for me as a person, he showed interest in me.” The patients and relatives felt both satisfied and secured by being treated at a distance.

In a few situations, the patients were surprised about how serious problems were taken. For instance, during one consultation a patient had signed up for a Midsummer Eve arrangement, but the experts found it necessary to rest the foot as much as possible and suggested a wheel chair. In the interview, the patient said that if he had not talked to the expert, “I would just have walked to the party.” A similar statement was given by the visiting nurse in the interview: “I would not have reacted towards the newly arisen blister and, furthermore, I would have treated the blister in a very different way than the one prescribed by the expert nurse.”

## 4. DISCUSSION

Our study indicates that treatment of diabetic ulcers in the home can be effectively supported by means of telemedicine. From the point of view of all three stakeholders (patient, visiting nurse, and expert), the simultaneous dialog facilitates satisfactory means for handling the cooperation and treatment.

To the experts, the communication with the patient in his own home environment brings to light personal issues enabling a better and more individual treatment. This may also have preventive effects as demonstrated in the case of the person attending the Midsummer Eve party. Contrasting indirect telemedical solutions, where digital images are obtained and forwarded to the expert by email (store-and-forward), our setup allows the participants to informally discuss, reflect upon, and solve discrepancies immediately in order for the treatment decision to be initiated straight away. Furthermore, the mobile setup makes it unnecessary to install and maintain equipment in the patient’s home.

While the studies by, for instance, Bowles and Dansky [16] investigate online connections between patient and expert directly, without the attendance of the visiting nurse who has the responsibility for carrying out the treatment between the expert consultations, our setup allows both the expert and the visiting nurse to participate actively in the expert consultations. This forges a synergetic combination of the different stakeholders’ competences. At the same time, the web-based online ulcer record facilitates a more traditional store-and-forward setup, and this study shows that the combination of these two methods is valuable in that the doctor and expert nurse could use the record for obtaining an overview of the situation and history, while at the same time collaborating with the visiting nurse and patient about the present situation.

As reported by others [8, 28], an important spinoff effect is that the competence of the visiting nurse may increase during this telemedical consultation facilitating the spread of knowledge from expert to the community.

In our study, the same team of clinicians conducted all 3 consultations with each patient, especially the patients experienced this to be a major advantage. This will not always be possible in a large-scale implementation. Thus, an important question to be investigated in future studies is the challenge of enabling experts to follow up on the basis of other persons’ descriptions and examinations.

It is also of vital importance that the initial visit to the outpatient clinic prior to the telemedical course of treatment should serve to get to know the patient in terms of the patient’s ability to participate in a course of treatment built on video consultations, which involves a thorough examination of the patient including measures such as peripheral blood pressure. Thereby, the patient obtains high quality and continuity in the treatment.

Both expert nurses and visiting nurses gained new roles in the telemedical setup. From the beginning of the study, the expert nurses were worried about not having the patient face to face and not having “the finger in the ulcer,” whereas the expert doctor tended to be more open to the concept, possibly because doctors are more used to delegating work tasks to others. The final focus group interview after the pilot test revealed that also the expert nurses were now confident with the new role as a consultant working through another nurse.

Communication through video phones puts constraints to the natural flow of the conversation in terms of delays and difficulties to interpret nonverbal cues and expressions. For this reason, all participants had to be conscious about their expressions and we recommend attention and training in this way of communication before implementing similar telemedical services.

The visiting nurse was invited into the experts’ reflections, which meant that her role went from carrying out the prescribed treatment on her own and without formal influence to be a fully fledged member of a collaborating team. Working through guidance from the experts, the visiting nurses became more engaged, secure, and responsible in carrying out the treatment. The competences and skills of the visiting nurse are likely to increase from each consultation, resulting in increased job satisfaction and qualifications.

## 5. CONCLUSION

In all consultations performed during our pilot test, the three criteria for the consultation to be characterized as a viable alternative to a visit in the outpatient clinic were met. The experts expressed satisfaction with their basis for decisions, the visiting nurses felt supported, and the patients experienced the new way of receiving treatment as an advantage. Furthermore, the simultaneous communication between expert, visiting nurse, and patient enabled the clinicians to provide a more holistic and tailor-made treatment, indicating that the telemedical consultations are a viable way of performing the treatment.
